# Supplementary material for: Are carbon emissions trading and green financial instruments synergistic? -Comprehensive quantitative research based on content analysis
Source: PLoS One. 2024 Mar 7;19(3):e0298601. doi: 10.1371/journal.pone.0298601 (PMC10919601; doi:10.1371/journal.pone.0298601)
Supplement: S1 Data — (ZIP) [file pone.0298601.s001.zip › Data/地级市Z_F工作报告.zip!/╒π╜¡/╓█╔╜╩╨/└┤╫╘╣1⁄2╓┌║┼í╛┬φ┐╦╩2╛▌═°í┐_2018.docx]

各位代表：

　　现在，我代表市人民政府向大会报告工作，请予审议，并请市政协委员和其他列席会议的同志提出意见。

　　一、2017年工作回顾

　　2017年是新一届政府的开局之年。市政府以习近平新时代中国特色社会主义思想为指引，深入贯彻党的十九大精神，全面落实省委、省政府决策部署，在市委的坚强领导下，在市人大、市政协的监督支持下，紧紧团结和依靠全市人民，激情创业，拼搏赶超，打好“五大会战”，建设“四个舟山”，实现经济社会平稳健康发展，较好地完成了市七届人大一次会议确定的各项目标任务。

　　（一）经济发展稳步提质增效。全年实现地区生产总值1219亿元，同比增长8.8%。完成财政总收入187.2亿元，其中一般公共预算收入125.8亿元，分别增长8%和10.6%。规模以上工业战略性新兴产业产值增长20.7%，高于规上工业总产值增速10.3个百分点。完成固定资产投资1450亿元，增长15%。168个省市重点项目完成年度投资830.5亿元，完成年度计划的110.9%。重大产业项目、高新技术产业、生态保护和环境治理投资分别增长56%、115.3%和87.8%。实际利用市外资金636.6亿元，增长24.7%；浙商回归省外到位资金234.9亿元，增长15%；实际利用外资4.05亿美元，增长92.8%。全市单位GDP能耗下降3.8%，节能减排各项指标完成省下达任务。

　　（二）重大战略加快落地实施。浙江自贸试验区总体方案获批，管委会挂牌运行，组建了国际咨询委员会和自贸试验区研究院。加快制度创新，复制落地前两批改革试点经验119项，争取国家和省级部门出台支持政策291条，形成制度创新成果和创新案例21项。浙江自贸试验区条例获省人大常委会审议通过。深化重大改革，建成自贸试验区综合服务大厅，开通6个“一口受理”综合窗口，对外资全面实施准入前国民待遇加负面清单管理模式。大力发展油品全产业链，突破保税油跨关区、跨港区直供等业务，出台国际航行船舶保税油经营管理暂行办法和业务操作规范，新批保税油经营资质企业5家，保税油供应量达到182.8万吨，增长71.8%；保税油结算量480.8万吨，占全国近50%。大力发展大宗商品贸易交易，成品油贸易交易突破1000万吨，保税油交易突破300万吨，天然气交易达到138万吨；混配矿突破800万吨，成为长江以南地区最大的混配矿基地。成功举办首届世界油商大会，签约重大项目20个，涉及总金额573.7亿元。区内新增企业4167家，注册资本2173亿元，企业平均注册资本大幅领先第三批其他自贸试验区。油品贸易企业大量集聚，浙江省石油公司注册落户。加快国际油品储运基地建设，黄泽山一期储罐项目完工，地下洞库和双子山项目前期工作有序推进。加快小干岛开发。启动舟山自由贸易港建设研究。舟山江海联运服务中心加速推进，累计投资突破500亿元，舟山港域江海联运量达到1.91亿吨。江海直达运输规范管理体系基本形成，填补国内空白。2万吨级江海直达首制船接水。国家交通运输公共信息平台江海联运信息中心获批运行。鼠浪湖40万吨矿石中转码头投入运行，铁矿石交易中心投入试运营。外供服务中心加快建设，供应货值突破4.5亿美元。全国首家海事法律服务中心成立运行。舟山绿色石化基地4000万吨/年炼化一体化项目通过环评和项目核准，一期项目全面开工。波音737完工和交付中心建设超常规推进，项目整体形象初步显现，舟山港综保区空港分区通过国家验收，舟山波音完工中心有限公司签约揭牌。中澳现代产业园澳牛进境加工项目开工建设。

　　（三）产业转型升级精准发力。工业发展稳步推进。召开全市工业发展大会，全面推进十类重大工业项目，完成工业投资450亿元左右。加快转型升级步伐，百项“机器换人”计划进展顺利，淘汰落后产能企业30家，整治低小散企业（作坊）170家，为企业减负22.8亿元。海洋电子信息、新能源、新材料等新兴产业加快发展，中船工业集团等一批央企和行业龙头企业注册落户，六横风电项目首台机组并网发电。成功纳入国家智慧海洋建设规划。获批省级军民融合创新示范区。现代服务业不断发展。全年港口货物吞吐量达4.58亿吨，占宁波舟山港的45%；金塘港区集装箱吞吐量突破100万标箱；普陀山机场旅客吞吐量首破100万人次。启动创建国家全域旅游示范区，成功举办2017国际海岛旅游大会，实现旅游总收入807亿元，增长21.9%。实现社会消费品零售总额506亿元，增长10.6%；网络零售额增长68.2%，增幅居全省第一。引导建筑和房地产业健康发展，商品住房库存消化周期缩短至7.3个月。引进租赁企业31家。渔农业加快转型升级。国家绿色渔业实验基地获农业部批复，国家远洋渔业基地获“国家科技兴海示范基地”称号，“中国鱿鱼交易中心”挂牌运行，建立全国首个海外远洋渔业流动基地，完成养殖塘生态化改造2310亩。全市渔业总产值达159亿元，增长7.4%；远洋渔业占渔业总产值的比重提升到32%。定海现代农业园区、普陀展茅田园综合体和3个特色农业强镇列入省级创建名单。普陀佛茶等一批农产品列入国家及省名特优新农产品目录。

　　（四）重点改革走在全省前列。深化“最多跑一次”改革，市级实现“最多跑一次”事项覆盖率达到97.7%，80%常办事项实现“办事不出岛”，取消社区证明153项。深入推进口岸通关机制改革，试点国际贸易“单一窗口”国家标准版，创造了船舶申报总量等3个全国第一。深化国资国企改革，组建市财金投资控股有限公司，城投和普发集团完成重组。出台企业上市挂牌三年行动计划，7家企业实质性启动上市工作。获批国家知识产权试点城市。加快建设特色小镇，嵊泗十里金滩小镇进入第二批省级特色小镇培育名单。推进渔农村产权制度改革，股改完成率达到99.2%，建立县、乡两级渔农村产权交易平台，全面完成三级渔农合联组建。36个乡镇（街道）基层治理体系“四个平台”建成运行，380个村（居）民委员会完成换届。探索实施聘任制公务员试点，在全省率先出台机关事业单位紧缺高端人才引进管理办法。完善人才政策体系，新引进海外院士1名、国家“千人计划”专家5名，国家“百千万人才工程”实现零突破，新引进“5313”领军人才（团队）26个。

　　（五）城乡建设管理统筹推进。加快城市设施建设。实施综合交通“1128”工程，加快开展甬舟铁路及高速公路复线前期工作，宁波舟山港主通道、普陀山机场改扩建、本岛定沈快速路顺利推进，新建停车泊位2673个，新增更新公交车100辆。实施海上花园城市建设攻坚行动，加快市政设施建设，大陆引水三期加快推进，新增地下空间74万平方米，建成综合管廊6.45公里，提标改造污水处理厂5座，新建污水管网62.1公里，建成城市公园、街头游园5个，新增绿色游步道140公里，新增绿化面积40万平方米。提升城市管理水平。理顺城市管理体制，成立市城市管理局。实施城乡环境综合整治十大行动，拆除违法建筑200多万平方米、户外广告牌1.5万块，取缔马路市场15个，关停并转废品收购站477家，248家无照经营点全部取缔。基本完成城中村改造18个，拆除10122户。改造完成C、D级城镇危房146幢和农村危房1084户。首批14个小城镇环境综合整治通过验收。深化美丽海岛建设。建成美丽海岛示范线5条、美丽渔农村5个、美丽家庭5000户，完成69个渔农村生活垃圾分类处理，嵊泗县成功创建省美丽乡村示范县。持续改善生态环境。全面实施《舟山市国家级海洋特别保护区管理条例》，出台全国首个海钓管理办法。打好“幼鱼资源保护战、伏休成果保卫战、禁用渔具剿灭战”，创新实施“滩长制”“湾长制”，放流鱼苗13.45亿尾（粒）。打好水气土污染防治攻坚战，剿灭426处劣Ⅴ类小微水体，县级以上集中式饮用水水源地水质全部达标，空气质量保持全国前列。嵊泗县获评首批省级生态文明建设示范县。

　　（六）社会民生事业持续发展。全市财政民生支出占一般公共预算支出的68.8%，增长4.6%。城乡居民收入分别增长8.5%和8.8%。十方面44项民生实事基本完成。“菜篮子”工作成效明显，基本品种价格与宁波市场差距控制在5%以内。打造“淘就业”平台，新增就业1.65万人，城镇登记失业率控制在2.7%以内。推进市区社保一体化改革，养老保险和医疗保险参保率分别达到86.72%和98.87%，异地就医实现全国联网结算。优化养老服务，新建居家养老照料中心20家，三级养老机构医养结合实现全覆盖。全面实施残疾人意外伤害保险政策，建成投运残疾人庇护性就业机构7家。完善住房保障体系，基本建成保障性住房6199套。率先在全省实现全域教育基本现代化，完成新高考改革试点，普惠性幼儿园覆盖面、义务教育标准化学校比例分别达到88%和95%，绿城育华学校建成开学，浙江国际海运职业学院岱山校区正式启用，新区旅游与健康职业学院波音员工培训中心加快建设，舟山职业技术学校（舟山技师学院）获评省首批“中职名校”。实施健康舟山2030行动，获批国家首批健康旅游示范基地，“双下沉、两提升”加快推进，“舟山群岛网络医院”建设受国办通报表扬，远程医疗服务超过25万人次。举办省第三届体育大会、2017舟山群岛马拉松赛，建成省级小康体育提升工程100个。渔农村文化礼堂社区覆盖率达70%，广电、新闻出版、档案、史志、文物和非遗保护等事业全面发展。认真做好信访工作，民族、宗教、国安、外事、侨务、老龄、儿童、红十字、慈善等事业取得新进步，工青妇等群团组织改革顺利推进。国防海防、民兵预备役、人民防空和双拥优抚等工作进一步加强。加强社会治安综合治理，圆满完成“平安护航十九大”工作，安全生产事故起数和死亡人数同比分别下降37.4%和20.9%，荣获全国综治最高奖“长安杯”和省“平安金鼎”。

　　（七）政府自身建设切实加强。注重加强政府系统思想和政治建设，推进“两学一做”学习教育常态化制度化，进一步增强“四个意识”。推进服务政府建设，加快实施“1113行动计划”，优化政务服务移动客户端平台功能，实现834项公共服务事项在线办理；推进新区“12345”平台与统一政务咨询投诉举报平台融合，完成政务服务网“一窗受理”系统，推动公共服务事项库、电子证照库、公共信用信息库开发。推进创新政府建设，充分发挥新区决策咨询委等智库作用，深化审计管理体制改革，率先推进“民生实事项目代表票决制”工作三级全覆盖，创新“自贸通才”培养机制。推进高效政府建设，推行“立等可取”“容缺受理”，市本级现场或当天办结率达88.9% ；实现“五小行业”一窗受理5个工作日内办结，自贸试验区外贸企业“证照联办”压缩至7个工作日。推进法治政府建设，提请市人大常委会审议地方性法规2件，制定出台政府规章2部、规范性文件87个；自觉接受人大、政协和社会各界监督，承办的223件人大代表建议、275件政协提案全部办复；完善府院联席会议制度，成立行政复议局；加强政府学法，在全省率先启动公务员“学法用法三年轮训”。推进廉洁政府建设，坚决贯彻落实中央“八项规定”精神，全面启用电子招投标系统，实现重大项目全程电子化招标，审计、监察全流程跟踪，一般公共预算“三公”经费支出同比减少8.3%。

　　各位代表，过去的一年，我们取得了一些成绩。这是党中央国务院关怀厚爱、省委省政府和市委正确领导的结果，是全市百万军民凝心聚力、共同奋斗的结果。在此，我代表市人民政府，向在各行各业辛勤劳动的全市人民，致以崇高的敬意！向人大代表、政协委员，向驻舟人民解放军和武警部队官兵，向在舟部省属单位，向各民主党派、工商联、人民团体和社会各界人士，向所有投资者、建设者，向关心和支持我市现代化建设的港澳台同胞和海内外朋友们，表示衷心的感谢！

　　回顾一年的工作，我们也清醒地看到，还存在不少的问题和困难：经济基础弱、总量小，落地和储备的大项目、好项目缺乏；新兴产业尚处于起步阶段，传统产业转型升级压力大，环境污染、产能落后、管理碎片化等问题制约高质量发展；综合交通等基础设施、公共配套设施建设水平滞后，城市规划建设管理与先进地区还存在不少差距，国际化短板十分突出；人才、资金等要素保障瓶颈依然明显；城乡、区域之间发展不够平衡，教育、医疗、养老等资源供给不够充分，与人民群众对美好生活的期望还有差距；政府职能转变还不到位，庸政、懒政、怠政现象还不同程度存在。对此，我们一定采取更加有效的举措，努力加以解决。

　　二、2018年总体思路和主要任务

　　2018年，是贯彻党的十九大精神的开局之年，是改革开放40周年，是舟山实现赶超发展、决胜高水平全面建成小康社会的关键之年。做好2018年工作，必须高举习近平新时代中国特色社会主义思想伟大旗帜，全面贯彻党的十九大精神，坚持稳中求进工作总基调，认真落实省委省政府和市委决策部署，按照“早日通高铁、插上金翅膀、牵好牛鼻子、做大油文章、加快国际化、争创自贸港”的要求，不忘初心、牢记使命，聚焦重点、全力攻坚，一以贯之打好“五大会战”，加快推动我市经济社会向高质量发展迈进，全面开创“四个舟山”建设新局面。

　　综合考虑宏观形势和我市经济社会发展实际，建议全市经济和社会发展的主要预期目标为：地区生产总值增长8%；一般公共预算收入增长8%；固定资产投资增长10%，其中交通投资、生态环境和公共设施投资、高新技术产业投资、民间投资均增长10%以上；社会消费品零售总额增长8.5%；城乡居民人均可支配收入分别增长8%和8.3%；节能减排降碳指标完成省定任务。

　　根据目标要求，重点做好五方面工作：

　　（一）聚焦开放水平抓国家战略。坚定不移打好系列国家战略落地大会战，全面实施“开放舟山”三年行动计划，积极融入“一带一路”、大湾区大花园大通道建设，主动接轨上海、宁波都市圈，加快把舟山建设成为在“一带一路”中居于枢纽位置的开放城市，争当我国全面开放排头兵。

　　全力建设自由贸易试验区。以挂牌一周年督查考核为契机，全力推进123项建设任务，全面复制推广前两批自贸试验区经验，形成制度创新成果和创新案例35项；进出口总额突破450亿元；铁矿石配矿贸易量力争达到1000万吨。大力发展油品全产业链，推进国际油品交易中心建设，推动大宗商品交易模式创新，加强与上海期货交易所合作，探索开展期现结合。推进国际海事服务基地建设，全面突破不同税号下保税油混兑，力争成为国内第一大加油港；加快发展船舶保税维修业务，做大做强船配交易市场，打造外供服务中心，外轮供应货值达到6亿美元。推进国际油品储运基地建设，加快推进黄泽山、双子山等离岛片区大型油品储运项目，全市形成2700万方储油能力。探索建设人民币国际化示范区，建立适合油品贸易的专用账户，推动油品等大宗商品现货贸易使用人民币计价结算。深化商事制度改革，全面实施“证照分离”改革试点。深化口岸通关便利化改革，全面启用国际贸易“单一窗口”。完善事中事后监管体系，出台外商投资等事中事后监管实施办法。加快落实首届世界油商大会成果，办好第二届世界油商大会。加快小干岛开发，开工建设自贸中心大厦、综合管廊设施等项目。力争突破首单飞机融资租赁业务。在加快建设自贸试验区的同时，全力争创自由贸易港。

　　加快建设舟山江海联运服务中心。建成投运光汇油品码头、实华二期、新奥LNG接收及加注站等一批重大项目，争取启动小洋山北侧集装箱内支线泊位、马迹山三期等项目，加快大洋山综合开发前期工作。实现港口货物吞吐量5亿吨，其中江海联运运量2.1亿吨。大力发展江海直达运输，完成商品汽车滚装等概念船型研发，组建江海联运船队。加快江海联运公共信息平台推广应用，完善信息互联标准规范，拓展航运电商等功能。加强与重庆、武汉、马鞍山等沿江港口城市合作，谋划推动一批合作项目落地。

　　加速建设国际农产品贸易中心。推进“一核心一平台四基地”建设，实现农产品贸易额160亿元。以国际高端动物蛋白加工贸易为核心，加快中澳现代产业园建设，打造金塘等国际农产品进口贸易加工示范区。以大宗商品交易中心为平台，拓展粮油、水产品等农产品上线品类，年交易量达到10万吨。建设国际高端动物蛋白冷链物流配送基地，完善冷链物流配送体系，拓展进口业务。建设国际远洋水海产品加工贸易基地，启动建设国际远洋水产品贸易中心，加快国家远洋渔业基地和国际水产城发展，做强“中国鱿鱼交易中心”品牌，培育围网金枪鱼交易中心，全年实现水产品贸易额65亿元。建设进境粮油保税交易加工基地，力争实现国际粮油交易中心基本运行，粮油贸易额达到60亿元。建设高端进口果蔬食品集散基地，发挥浙台经贸合作区作用，深化与台湾及东南亚国家和地区贸易合作。

　　（二）聚焦高质量抓创新发展。全面实施“创新舟山”三年行动计划，推动经济发展质量变革、效率变革、动力变革，不断增强我市经济创新力和竞争力。

　　创新项目推进机制。全力打好招商引资大会战，全面推进“市县长项目工程”，持续实施“2553”计划，争取引进市外资金660亿元，浙商回归省外到位资金235亿元，实际利用外资5亿美元以上。完善项目中心制，大力推进161个市重点项目建设，完成投资684亿元。强化要素保障，积极争取耕地跨省占补国家统筹政策，科学开发海域海岛资源，探索破解环境容量制约的有效途径。

　　全力攻坚产业创新。大力发展四大千亿和若干百亿产业，构建现代化海洋经济体系。加快建设绿色石化基地，力争一期项目年底建成。高质量推进波音737完工和交付中心项目，确保年底交付首架飞机。力争波音全球供应商大会在舟山召开，拓展航空高端制造和配套服务产业链。推动船舶与海工装备业智能化生产改造与应用，加快绿色节能、高技术高附加值船舶和海洋工程装备研发制造，突破军船及辅助船制造、维修，加快重点船企重整重组。推进国家全域旅游示范区创建工作，争创全国邮轮旅游发展实验区，谋划申报国际旅游岛，旅游总收入突破900亿元，旅游业增加值占全市GDP比重达8.5%以上。发展国际海事服务业，建设新城航运服务集聚区，积极推进燃供企业结算中心落户，培育海事金融、保险、信息、仲裁等服务业。推动水产品加工、机械汽配制造、螺杆等特色产业向“高新优”方向发展。建设国家智慧海洋产业示范区，全面落实产业培育实施方案，推进智慧海上国防动员平台等项目。发展大健康产业，建设国家健康旅游示范基地，引进高端特色健康旅游服务项目，支持普陀大健康产业园建设。加速金融集聚区建设，完善融资租赁业发展政策扶持体系，加快民营银行筹建步伐。加快现代渔业发展，建设国家绿色渔业实验基地，推进海洋经济创新示范城市建设，开展渔业产权化改革，实施海洋牧场、渔船和装备提升等十大工程，促进休闲渔业转型升级。

　　提升科技创新水平。全力创建国家高新技术产业开发区。大力发展科技企业，新增高新技术企业40家，培育科技型中小微企业144家。大力促进科技成果转化，实现技术成果交易额4.5亿元以上。加快国家知识产权试点城市建设。实施“高精尖”海洋创新人才引进计划，引进培育20人以上。深入实施“5313”行动计划升级政策，引进科创领军人才（团队）企业20家。落实鼓励大学生来舟就业创业政策，壮大基础人才队伍。

　　（三）聚焦市场活力抓重大改革。坚持以供给侧结构性改革为主线，深化简政放权，加快重点领域和关键环节改革，完善法治化、国际化、便利化营商环境，不断激发市场活力和社会创造力。

　　深化供给侧结构性改革。坚持“亩均论英雄”，完善要素市场化配置，建立产业结构调整创新、民间资本吸纳等体制机制。推进小微企业园区建设，开展“低小散企业”专项整治，加快“僵尸企业”市场出清。实施“机器换人”“智能制造”项目100项以上。推进“三强一制造”工作，推动企业加强质量提升示范和品牌培育。促进建筑业和房地产业平稳发展，商品住宅价格、去化周期控制在合理范围。推动重大项目、重点领域信贷投放，增加金融有效供给。

　　做实“最多跑一次”改革。全面推进“最多跑一次”改革标准化、规范化建设，力争年底前覆盖所有民生事项和审批事项，其中50%以上的民生事项实现“一证通办”。全面打破信息孤岛，以政务服务网为平台加快公共数据整合共享，推进政府数字化转型。推进“标准地+承诺制”改革，实施“区域能评、环评+区块能耗、环境标准”制度。实现一般企业投资项目开工前审批“最多跑一次”、最多100天，力争实现竣工验收前“最多跑一次”。推动涉政中介机构市场化改革，提升中介服务效率和水平。深化商事登记领域改革，统筹推行“多证合一、证照联办”。攻坚“办事不出岛”，基层便民服务中心全部实现“一窗受理”。深入开展多部门、多层级“一件事”梳理工作，推动“一件事”一窗办。完善统一政务咨询投诉举报平台，提高群众诉求一次性解决率。

　　加快推动重大领域改革。强化市级统筹，深化城市规划建设管理等领域市区一体化改革。实施“凤凰行动”，加大国有企业资产证券化力度，新增上市挂牌企业4家以上。加强金融风险防范，深化互联网金融整治，严密防控关注类贷款比例和不良贷款率。加快13个省市特色小镇建设。争创国家军民融合创新示范区。探索产业创新综合体建设。

　　（四）聚焦品质提升抓环境优化。全面实施“品质舟山”三年行动计划，加快推动城市面貌“一年一个样、五年大变样”，打造最干净最美丽的海上花园城。

　　提升城市国际化水平。塑造国际花园城形象，启动城市总体规划局部调整，编制定海、普陀、新城分区规划，加强城市建设管控和引导，推进定海“花园式国际人文港城”、普陀“海上花园会客厅”、岱山“全域全景海上花园城”和嵊泗“美丽海岛 花园微城”建设，聚力打造新城“中国海上花园城市样板区”。提升城市通达水平，打好综合交通建设大会战，加快推进甬舟铁路及高速公路复线、舟山至上海跨海大通道前期工作，加速宁波舟山港主通道等工程建设，如期建成鱼山大桥。加快普陀山机场改扩建工程，完成国际航站楼一期建设。加速城市中部快速通道建设，启动城市轨道交通建设方案策划研究。提升建设18座陆岛交通码头，开通水上飞机航线。打造宜居宜业宜游的国际化环境，加快国际化街区、国际化社区建设。探索国际化合作办医、办学、办赛机制。实施自贸区人才签证制度。培养多语种行业服务人才。加快双语标识标牌改造。加强国际友好合作，办好“一带一路”国际海岛旅游发展舟山论坛等国际会议，做大国际海岛旅游博览会等国际会展，办好世界海洋日活动暨中国海洋文化节，做精舟山群岛马拉松、国际自行车赛等赛事，密切与国际友好城市的交流合作。

　　加快城市高品质发展。完成32个城中村治理改造任务，加强拆后土地规划利用。打好城乡环境综合整治大会战，重点实施城区停车秩序、交通隔离设施等整治行动。推进“三改一拆”和小城镇综合整治，拆除违法建筑100万平方米，力争完成20个小城镇达标验收，创建“基本无违建市”。完善城市基础设施建设，大力推进16个城市公园建设，打通、拓宽断头路、瓶颈路12条，新增城区公共停车泊位1500个。推进厕所革命，新、改建公厕120座。打造城市核心景观带，加快百里滨海大道建设，美化滨海廊道沿线景观，新增城市绿道20公里、公共绿地10万平方米。改造提升城市入城口8个，打造精品示范街（路）10条，继续推进中心城区、重要节点景观亮化。出台市容市貌、园林绿化等管理制度。加快智能城市建设，建成投用城市管理“一中心四平台”。

　　全面实施乡村振兴战略。编制好乡村振兴战略规划，实施美丽乡村建设三年攻坚行动，启动建设2个美丽小岛和4个乡村振兴战略示范村，打造4条美丽景观线，创建18个美丽精品村，提升500家精品民宿，培育3900户美丽庭院。实施美丽农业发展攻坚行动，推进省级现代农业园区和田园综合体发展，抓好重点农业乡镇建设，提高粮食保障水平，新认证绿色食品基地2000亩。发展休闲景观农业，打造本岛北向疏港公路沿线景观农业示范带。发展壮大集体经济，力争全市三分之一以上渔农村社区集体经济年收入达到30万元以上，全面消除集体经济薄弱村。开展农村人居环境整治三年行动，推进整洁家园、整洁田园、整洁水源等专项整治。深化农村土地和集体产权制度改革，引导土地经营权流向新型农业经营主体。

　　打造更优质生态环境。创建国家生态文明建设示范区。打好治水治污大会战，实施“品质河道·碧水行动”三年计划，固化剿劣治水防反弹长效机制，深化河长制，创建“污水零直排区”；全面实施污水处理厂提标改造，开工建设舟山市污水处理中心，新建、改造污水管网72公里。深化研究引进大陆优质水源方案，推进本岛水系连通、库库联网，开展全国海水淡化试点工作。加快工业固废、危险废物处理设施建设，实现危废处理全覆盖。加大大气污染治理力度，深化挥发性有机物治理。全面推进生活垃圾分类处理，加快垃圾焚烧发电项目三期建设，城市生活垃圾无害化处理率达到99%，建成渔农村生活垃圾分类处理村88个。创建国家森林城市和国家园林城市，实施森林抚育2.1万亩，建设彩色健康森林5000亩，完成破损山体修复17.44公顷，治理废弃矿山15处以上。加强近岸海域污染整治，深化滩长制，实施海洋生态整治修复工程，整治修复海岛岸线30公里。

　　（五）聚焦幸福导向抓民生改善。全面实施“幸福舟山”三年行动计划，补短板、促均衡、提质量，持续增强人民群众获得感、幸福感、安全感和自豪感。

　　共创文明家园。深化全国文明城市创建，认真实施《舟山市文明行为促进条例》，培育践行社会主义核心价值观，争创国家社会信用体系建设示范城市。优化公共文化设施布局，推进市海洋文化艺术中心二期、定海图书馆、普陀美术馆等建设，新增城市书房5家、小城镇文体休闲广场（公园）6个。完善渔农村文化礼堂长效机制，新建文化礼堂18家。实施文化惠民工程，全面升级“淘文化”公共服务平台，推动各类公共文化设施向公众开放。推进优秀传统文化传承发展，加强文化遗产保护。倡导全民健身、全民阅读，提升全民文化素养。

　　共建平安家园。深入推进“雪亮工程”，全面升级立体化社会治安防控体系，完善基层治理体系“四个平台”，探索建立新区社会动员体系和社会心理服务体系，优化矛盾纠纷多元化解机制，打造全国最安全城市。完善安全生产体制机制，落实企业主体责任，抓好重点领域专项整治，完善防灾减灾体系建设，坚决遏制重特大安全事故发生。继续推进“无欠薪市”创建、“最安全旅游岛（景区）”创建、“两非”车辆专项治理等工作。

　　共享幸福家园。坚持示范引领，新增幸福社区34个。大力推进就业创业，完善智能化人力资源市场平台建设，升级打造“淘就业”品牌。新增城镇就业1.2万人，城镇登记失业率控制在3%以内。推动社保基金市区一体化运行，基本养老保险和基本医疗保险的参保率分别稳定在86%和98.5%以上。关注低收入困难群体，开展低收入渔农户十大帮扶行动，实现80%重点帮扶的低收入家庭年人均纯收入达到1.2万元以上。构建多元化养老服务体系，推进医养护一体化，鼓励社会力量参与，建成示范型居家养老服务照料中心5家。发展残疾人事业，加强残疾预防和康复服务，新建残疾人庇护性就业机构5家。深化教育综合改革，稳妥实施中考改革，理顺市、区高中教育和学前教育管理体制，确保特色示范高中比例达到80%以上。实施中心城区中小学（幼儿园）专项规划，开展新一轮学前教育发展行动，建成小学3所、幼儿园4所，新建初中1所。积极创建国家义务教育优质均衡县（区），推行新城义务教育集团化办学，加大偏远海岛地区教育扶持力度。促进职业教育产教融合和校企深度合作，加强高等教育统筹管理和校地融合发展。推进健康舟山建设，顺利通过国家卫生城市第二轮复评。深化医药卫生体制改革，完善“双下沉、两提升”长效机制，建设高水平的区域医疗联合体。深化“舟山群岛网络医院”应用，实现有基层医疗机构的岛屿全覆盖。健全基层医疗服务体系，开展责任医生签约服务精细化试点，加强全科医生培养。出台鼓励生育二孩的地方性政策，实施妇女儿童健康关爱工程，建成规范化母婴室50个。扎实推进援疆援藏援青援川工作，加强与松原市对口合作。大力发展红十字、慈善、志愿服务等公益事业，支持工会、共青团、妇联、科协等群团改革与发展。支持驻舟部队建设，抓好国防动员和后备力量建设，推动双拥工作创新发展。

　　各位代表，今年，我们继续按照民生实事“群众提、大家定、政府办”的理念，经过广泛征集和充分讨论，梳理形成了市政设施、教育发展、医疗卫生、社会保障等方面24项候选项目，提请各位代表票决。我们将根据票决结果，认真组织实施，确保全面完成，让全市人民共享发展成果。

　　各位代表，做好2018年各项工作，必须以“不忘初心、牢记使命”主题教育为主抓手，全面加强政府自身建设。新的一年，我们提高站位，聚焦系列国家战略落地实施，做好“十三五”规划中期评估工作，集中力量谋路径、抓落实，以更大的气魄、更宽的视野、更高的标准，全力提升推动改革发展的能力。我们依法行政，健全重大事项决策前向人大报告制度，充分发挥政协民主协商作用，自觉接受监督，加强政府内部审计，全力提高履职能力和履职科学化水平。我们实干为民，牢固树立“群众利益无小事”理念，聚焦群众关切，攻坚民生短板，让所有工作都充分体现人民意愿、维护人民利益、接受人民监督，以实实在在的工作和成效，全力赢得党和人民的信任。我们廉洁奉公，牢记作风建设永远在路上，巩固拓展落实中央“八项规定”精神成果，持之以恒正风肃纪，全面营造政府系统风清气正的政治生态，全力打造一支激情创业能打胜仗全面过硬的新区铁军。

　　各位代表，新时代要有新作为，新作为更需新担当。让我们紧密团结在以习近平同志为核心的党中央周围，在省委省政府和市委的坚强领导下，紧紧依靠全市人民，紧紧围绕“自由贸易港、海上花园城”目标，干字当头，接力攻坚，为加快“两个高水平”建设、实现全市人民对美好生活的向往而努力奋斗！
